# Mr. Scott, on the Virtues of Spider's Web

**Published:** 1809-08

**Authors:** James Scott

**Affiliations:** Surgeon. His Majesty's Ship Euryalus, Downs


### *To the Editors of the Medical and Phyſical Journal.*

GENTLEMEN,

IN Number 125 of your Journal, I observe with pleasure, under the head of "Correspondence," what I cannot help thinking extremely proper, namely, a Reply to an Anonymous Writer, who ventured to offer remarks on a communication which you received with its author's real signature. Indeed, I have always considered those papers which are only signed with initials, or some convenient word, as "Crito, Verax, &c." not to be written with that purity of intention, or strict adherence to truth, which should be paramount to all other considerations in every thing relating to a science so intimately connected with the happiness of mankind; and although my opinion, universally applied, may be rather harsh, yet I own I should be pleased to find that such communications were uniformly rejected.

In the instance alluded to, although you have not mentioned the name of the author on whose paper the anonymous writer commented, I believe Dr. Jackson's Observations on the Virtues of Spider's Web, were the subject of his censure; and from his subscribing himself "An Essex Practitioner," I doubt not he is the person who, in Number 124, informs us of the great mischief he has seen occasioned in many cases, by the administration of arsenic in intermittent fevers, and that he expects to "see many more, as it is become extremely common for "Lady Doctors," and other persons who are not in the profession, to dispense the arsenic solution with a very liberal hand to the poor and needy."

His dislike to female and interloping Practitioners, I cannot altogether disapprove of, but I fear his zeal for the honour of the profession, and the interest of its regular members, may have led him too far into the practice of condemning every remedy which looks like an old woman's nostrum.

It is however certain, that many medicines which have failed in their intended effects, in the hands of regular Esculapians, have been administered with the most flattering success by Quacks and Female Physicians; and to what can we attribute the failure of the former, but to a very reprehensible timidity in the exhibition of medicines, which I am persuaded is the chief reason that digitalis, and other invaluable articles have fallen so low in the general estimation of the medical world? Under that conviction I

have given fox-glove in substance, in pneumonia and other acute diseases, in doses of 2, 3, 4, and even 5 grains, repeated two or three times a day; or according to its effects on the pulse; and I believe that after a large bleeding, as ℔j. or 20 ounces, I have frequently seen the repetition of venesection rendered unnecessary by 2 grains of the powder.

As a vulnerary, spider's web has long stood high in the opinion of the vulgar, not merely on account of its styptic virtue, but because of an anodyne quality which they suppose it to possess in an eminent degree; for which reason it is frequently applied to painful and ill-conditioned wounds and ulcers; and in such cases the "Lady Doctors" (who are often as successful as the regulars in simple complaints) assert that it produces the best effects. Indeed, when we consider the well known efficacy of some animal drugs, as castor and musk, I do not see any reason to doubt the virtues which spider's web is said to possess; and although musk and castor might naturally be expected to exert considerable effects on the system, in consequence of their strong and peculiar odour and taste, yet the absence of those sensible qualities in spider's web, should by no means be considered even a presumptive evidence of its medicinal inefficacy; for, as an elegant and learned writer* has observed, substances do not affect the body in proportion to the quantity of what we denominate their active qualities, but according to laws widely different. A grain of the white oxyd of arsenic does not impress the organs of taste, as being peculiarly acrimonious, nor does any chemical test prove that it contains more of the principle of astringency than a drachm of the powder of galls; yet, variously administered, this apparently inconsiderable quantity can destroy life or cure disease.† I will therefore take it for granted that spider's web, though tasteless, and almost inodorous, has medicinal virtues; and if the modus operandi of this remedy cannot be satisfactorily accounted for, according to the generally received theory of intermittent fevers, it must also be allowed, that our pathological disquisitions are clouded with so much uncertainty, that we cannot positively say we are perfectly acquainted with the action of any medicine.

But it is plain to me, by Dr. Jackson's testimony, that cobweb did arrest the paroxysms of intermittents in his patients, and that too under circumstances where the imagination

* Dr. Edmondston.

† Dr. Edmonston's "Treatise on the Varieties and Consequences of Ophthalmia," p. 44, (Note.)

tion was unimpressed by any particular expectations; and I have lately been informed of a case of ague in which a cure was effected by the patient swallowing a spider, after all the usual remedies had failed. In this instance, the spider was had recourse to without the advice of medical attendants, and, indeed, before Dr. Jackson wrote on the subject.

The patient was a young lady who had a particular aversion to spiders, and was literally forced to swallow the insect in a spoonful of currant jelly; of course she was acted upon, not by the exhilarating hope of receiving benefit, but by fear and disgust, which being depressing passions, could only operate as sedatives; yet the remedy was effectual; and if the paroxysms of intermittents are cured by stimulants, the cobweb must have been concerned in the sanative process.

The "Essex Practitioner" may perhaps wish to know further particulars of this case; and if so, I will readily communicate them, but at present I shall content myself with observing that I have the pleasure of the lady's acquaintance; and when I pledge my honour to the truth of my assertions, I conceive it unnecessary to mention the fair patient's name or residence, as it might not be altogether pleasing to her to appear before the public.

Since Dr. Jackson's paper was published, I have had no occasion to employ the remedy he proposes, but I shall most anxiously embrace the earliest opportunity to give it a fair trial.

In the mean time, I hope you may be less troubled by anonymous Correspondents, and that candour and sincerity may be a prominent feature in all medical communications.

I am, &c.

JAMES SCOTT, Surgeon.

*His Majesty's Ship Euryalus, Downs,*

*July* 9, 1809.

